# Defective MOFs Microreactor with Heterojunction for Selective Methane Photocatalysis via Desorption‐Driven Overoxidation Suppression

**DOI:** 10.1002/advs.202513507

**Published:** 2025-10-27

**Authors:** Bo Feng, Danning Feng, Kun Wan, Yan Pei, Baoning Zong, Hexing Li, Wei Li, Minghua Qiao

**Affiliations:** ^1^ Department of Chemistry Shanghai Key Laboratory of Molecular Catalysis and Innovative Materials State Key Laboratory of Porous Materials for Separation and Conversion Fudan University Shanghai 200438 P. R. China; ^2^ State Key Laboratory of Petroleum Molecular and Process Engineering Research Institute of Petroleum Processing SINOPEC Beijing 100083 P. R. China; ^3^ Key Laboratory of Resource Chemistry of Ministry of Education Shanghai Key Laboratory of Rare Earth Functional Materials, College of Chemistry and Materials Science Shanghai Normal University Shanghai 200234 P. R. China

**Keywords:** carbon nitride, copper single atoms, MOFs, heterojunction, oxidation of methane

## Abstract

Photocatalytic oxidation of methane to methanol oxygenates (CH_3_OH and CH_3_OOH) under mild conditions represents a promising approach for methane valorization, yet achieving both high efficiency and high selectivity remains a significant challenge. Herein, a defect‐engineered spatial coupling strategy is devised to construct a heterojunction photocatalyst (Def‐CuCN/NU) by integrating copper single atoms (Cu SA) anchored on polymeric carbon nitride (CN) into the defective NH_2_‐UiO‐66 (Def‐NU). The defective MOFs with porous structure and abundant active sites serve as microreactors that enhance methane adsorption and promote methanol desorption, thus promoting the reaction and minimizing the contact of methanol with ·OH radicals and effectively suppressing overoxidation. Additionally, the heterojunction formed between CuCN and Def‐NU accelerates charge separation and transport, which renders efficient photocatalytic conversion of methane to methanol oxygenates. Notably, the Def‐CuCN/NU catalyst affords a high production rate of 1718 µmol g^−1^ h^−1^ for methanol oxygenates under full‐spectrum light irradiation at a remarkable selectivity of 96.5%. This study presents the first demonstration of employing defect‐engineered MOFs‐based heterojunction photocatalysts for the regulation of the reaction pathway to enable highly selective photocatalytic oxidation of CH_4_.

## Graphical abstract

A defect‐engineered MOFs microreactor is constructed to regulate product desorption to suppress overoxidation by integrating Cu single atoms anchored on polymeric carbon nitride into the defective NH_2_‐UiO‐66. In photocatalytic oxidation of CH_4_, Def‐CuCN/NU enhances CH_4_ adsorption and CH_3_OH desorption, while the type‐S heterojunction enhances charge separation, rendering an excellent CH_3_OH oxygenate yield of 1718 µmol g^−1^ h^−1^ and selectivity of 96.5%.

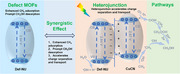

## 1 Introduction

Methane (CH_4_), the main component of natural gas, is an abundant and inexpensive carbon feedstock, and its selective oxidation into value‐added oxygenates holds significant economic and environmental importance.^[^ ^1^, ^2^, ^3^ ^]^ The activation of CH_4_ under mild conditions remains challenging due to its highly symmetrical structure and strong C─H bonds (439 kJ mol^−1^), with industrial methods typically requiring harsh conditions that result in high energy consumption and safety problems.^[^ ^4^, ^5^ ^]^ Photocatalysis has recently emerged as a promising alternative, enabling CH_4_ conversion under ambient conditions by utilizing solar energy over semiconductor materials.^[^ ^6^, ^7^, ^8^ ^]^ Progress has been made in converting CH_4_ to valuable liquid oxygenate products, such as methanol (CH_3_OH),^[^ ^9^, ^10^ ^]^ methyl hydroperoxide (CH_3_OOH),^[^ ^11^, ^12^ ^]^ formaldehyde (HCHO),^[^ ^13^, ^14^ ^]^ formic acid (HCOOH),^[^ ^15^, ^16^ ^]^ acetic acid (CH_3_COOH),^[^ ^17^, ^18^ ^]^ and ethanol (C_2_H_5_OH).^[^ ^19^, ^20^ ^]^ However, achieving both high efficiency and high selectivity for a specific product while avoiding overoxidation remains a significant challenge.

Metal−organic frameworks (MOFs), composed of metal clusters and organic linkers assembled into crystalline porous structures, have been extensively adopted for the precise design of photocatalysts due to their highly tunable architectures, rich coordination environments, and well‐defined microporous channels.^[^ ^21^, ^22^, ^23^ ^]^ MOFs are considered as promising ‘microreactors’ for photocatalytic methane oxidation, as the porous structures provide abundant active sites and spatial confinement effects.^[^ ^10^, ^24^ ^]^ However, MOFs still face the following issues in practical photocatalytic applications. On the one hand, MOFs assembled from organic ligands possess narrow micropores that greatly hinder substrate access and products diffusion.^[^ ^25^, ^26^ ^]^ Delayed desorption of products results in prolonged contact between CH_4_ oxidation products and hydroxyl radicals (·OH), thus causing overoxidation to HCHO and even CO_2_. For example, previous studies have shown that the microreactor environment within the pores of MOFs can enhance the photocatalytic oxidation of CH_4_ by facilitating the rapid coupling of ·CH_3_ and ·OOH radicals, but with the overoxidation product HCHO being predominant.^[^ ^24^ ^]^ Constructing a more open pore architecture that enables rapid product desorption is an effective approach to avoid overoxidation. Various strategies have been employed to expand the pore structures of MOFs, including the incorporation of elongated linkers,^[^ ^27^, ^28^ ^]^ modulator‐assisted synthesis,^[^ ^29^ ^]^ ligand exchange,^[^ ^30^ ^]^ chemical etching,^[^ ^31^, ^32^ ^]^ and the introduction of defects via ligand pyrolysis.^[^ ^33^, ^34^ ^]^ Defective MOFs synthesized via ligand pyrolysis not only retain their crystalline structure but also develop mesoporous channels while preserving the modified groups.^[^ ^35^, ^36^ ^]^ On the other hand, MOFs suffer from low photogenerated charge separation efficiency and rapid electron–hole recombination.^[^ ^37^ ^]^ An effective strategy to overcome these shortcomings is to construct heterojunctions with graphitic carbon nitride (CN),^[^ ^38^, ^39^, ^40^ ^]^ a material that features suitable band gap, high stability, low toxicity, and facile synthesis.^[^ ^41^, ^42^, ^43^ ^]^ Moreover, recent studies have highlighted the significant advantages of incorporating copper single atoms (Cu SA) onto CN for photocatalytic CH_4_ oxidation to valuable liquid products.^[^ ^44^, ^45^, ^46^ ^]^ Therefore, it is anticipated that a combination of Cu SA, CN, and defective MOFs to form a composite heterojunction material holds great promise for achieving highly efficient photocatalytic CH_4_ oxidation while effectively mitigating overoxidation due to the following three key merits. First, the defect‐engineered MOFs microreactors exhibit excellent CH_4_ adsorption capacity and facilitate product diffusion to avoid prolonged residence in the pores and prevent overoxidation. Second, the heterojunction structure effectively promotes the separation of photogenerated electron−hole pairs, thus significantly improving photocatalytic efficiency. Third, the introduction of Cu SA sites facilitates the selective activation of O_2_ to ·OOH species rather than the highly oxidative ·OH radicals, thus further suppressing CH_4_ overoxidation.^[^ ^24^ ^]^ However, as far as we are aware of, there is no study on the use of defective MOFs composite heterojunction materials as microreactors to regulate rapid product desorption and suppress overoxidation in photocatalytic CH_4_ oxidation.

**Scheme 1:**
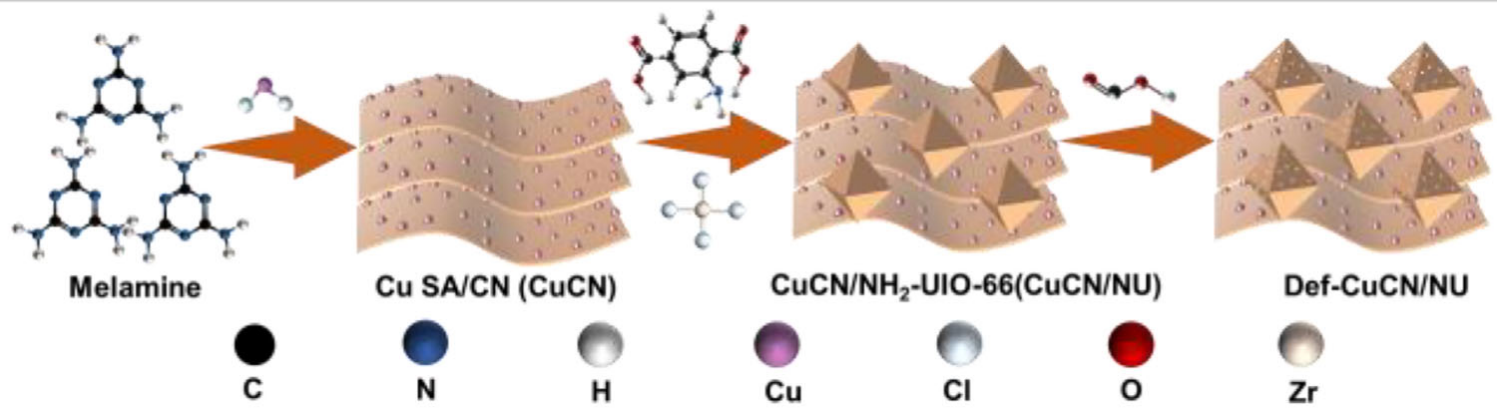
Schematic diagram of synthesis of Def‐CuCN/NU.

In this work, we report a defect‐engineered MOFs constructed by intercalating Cu SA anchored on polymeric carbon nitride (CuCN) into the defective NH_2_‐UiO‐66 (Def‐NU) microreactors. The synthetic process is depicted in **Scheme** **1**. The resulting Def‐CuCN/NU heterojunction catalyst exhibits excellent CH_4_ adsorption capacity, enhanced separation and transport of photogenerated charge carriers, and rapid product desorption within the microreactor. Under full‐spectrum irradiation for 3 h, the catalyst achieved a total yield of 51.6 µmol of methanol oxygenates (CH_3_OH and CH_3_OOH), corresponding to a production rate of 1718 µmol g^−1^ h^−1^, and selectivity of 96.5%. This study presents the first report of defect‐engineered MOFs‐based heterojunction as microreactors for photocatalytic CH_4_ oxidation, effectively addressing the issue of overoxidation caused by delayed product desorption.

## 2 Results and Discussion

### 2.1 Physical Characteristics

**Figure 1:**
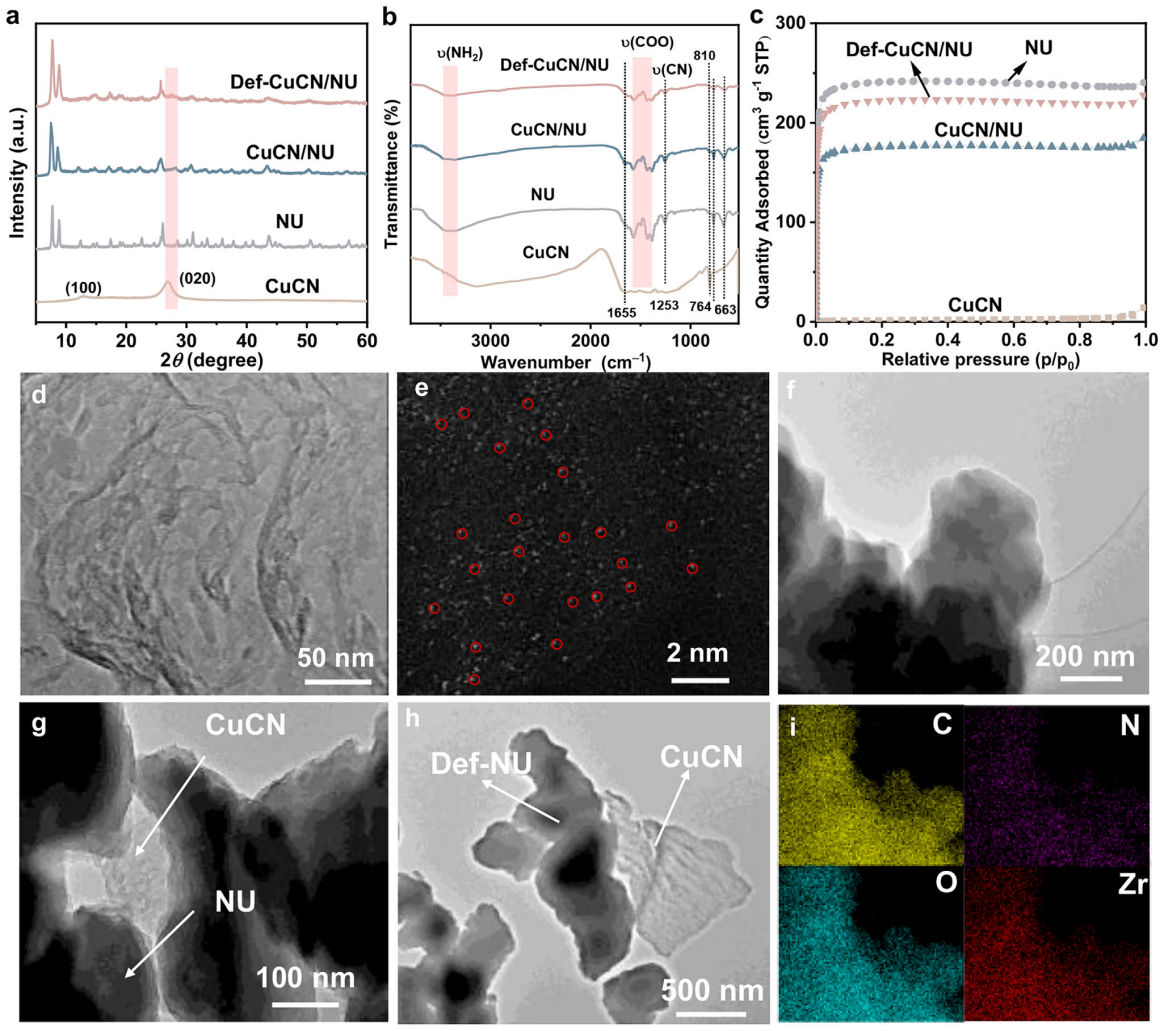
a) XRD patterns, b) FTIR spectra, and c) N_2_ adsorption–desorption isotherms of CuCN, NU, CuCN/NU, and Def‐CuCN/NU. TEM images d,e) AC‐HAADF‐STEM image of CuCN. TEM images of f) NU, g) CuCN/NU and h) Def‐CuCN/NU. i) EDS mapping images of Def‐CuCN/NU.

X‐ray diffraction （XRD）patterns of the CuCN, NU, CuCN/NU, and Def‐CuCN/NU nanocomposites are presented in **Figure** **1**a. The peaks at 12.7° and 26.9° are characteristic of the (100) and (002) diffractions of CN, respectively,^[^ ^47^, ^48^ ^]^ and NU exhibits the characteristic diffraction peaks of NH_2_‐UiO‐66.^[^ ^49^ ^]^ The absence of Cu‐related peaks indicates that Cu species are highly dispersed within the carbon nitride framework. The coexistence of the diffraction peaks of CuCN and NU in the CuCN/NU composite confirms the successful formation of the heterostructure. Moreover, the shift of diffraction peaks of NU toward lower angles (Figure S1a, Supporting Information) suggests the uniform dispersion of CuCN and lattice expansion of the Zr─O clusters within the NU framework.^[^ ^50^, ^51^, ^52^ ^]^ To endow NU with enriched pore structures, CuCN/NU was subjected to vacuum treatment at 200 °C to induce defect formation, yielding defect‐structured CuCN/NU (Def‐CuCN/NU). It is observed that the diffraction peaks of Def‐CuCN/NU are similar to those of CuCN/NU, indicating that the MOF structure is preserved, while the broadening and up‐shift of the peaks at 7.7° and 8.7° (Figure S1b, Supporting Information) imply a reduction in both the crystallite size and intercluster lateral distance.^[^ ^33^, ^53^, ^54^ ^]^ When the treatment temperature is elevated to 250 °C, Def‐CuCN/NU becomes amorphous (Figure S1c, Supporting Information), indicating the collapse of the MOFs at this temperature.^[^ ^55^ ^]^

Fourier‐transform infrared (FTIR) spectra in Figure 1b show that the synthesized NU exhibits peaks characteristic of NH_2_‐UiO‐66, where two major amine characteristic peaks at 3468 and 3370 cm^−1^ are attributed to the asymmetric and symmetric stretching vibrations of the N─H bond.^[^ ^56^, ^57^ ^]^ Additionally, the N─H wagging vibration is confirmed at 764 cm^−1^. The peaks at 1579 and 1384 cm^−1^ are assigned to the asymmetric and symmetric stretching vibrations of the COO^−^ group.^[^ ^58^ ^]^ The characteristic peak of the C─N bond in the organic ligand is observed at 1260 cm^−1^,^[^ ^59^ ^]^ and bending vibrations of ─OH and C─H give the band at 663 cm^−1^.^[^ ^58^, ^60^ ^]^ The peaks of the CuCN/NU composite are similar to those of NU, due to vibrational overlapping between CuCN and NU. For Def‐CN/NU, the FTIR spectrum revealed the removal of the guest molecule DMF, as evidenced by the weakening of its characteristic peak at 1655 cm^−1^.^[^ ^59^ ^]^ The reduction in the intensities of the COO^−^ stretching bands may signify either the dehydration of bridging *μ*_3_‐OH groups in the Zr_6_O_4_(OH)_4_ clusters or a decarboxylation process.^[^ ^33^, ^58^ ^]^ The preserved N─H and C─N vibrations clearly indicate that the structure of Def‐CuCN/NU remains intact and the ─NH_2_ groups are retained after thermal treatment. The ─NH_2_ groups are capable of modulating the band structure, narrowing the band gap, and promoting the generation and separation of photogenerated charge carriers.^[^ ^33^ ^]^

The N_2_ adsorption isotherms are presented in Figure 1c. As summarized in Table S1 (Supporting Information), the Brunauer−Emmett−Teller specific surface area (*S*_BET_) decreases from 841 m^2^ g^−1^ for NU to 519 m^2^ g^−1^ for CuCN/NU, indicating successful integration of CuCN nanosheets with NU and partial pore blockage that reduces N_2_ adsorption. The introduction of defects to CuCN/NU leads to a significant increase in the *S*_BET_ to 749 m^2^ g^−1^, along with notable enlargements in pore volume from 0.24 to 0.35 cm^3^ g^−1^ and pore diameter from 2.4 to 3.2 nm, ascribable to the generation of abundant porous structures within the NU due to linker pyrolysis. It has been reported that MOFs with highly porous structure can serve as microreactors for efficient photocatalytic oxidation of CH_4_.^[^ ^10^, ^24^ ^]^

Scanning electron microscopy (SEM) and transmission electron microscopy (TEM) images show that CuCN exhibits a typical nanosheet morphology (Figure 1d; Figure S2a, Supporting Information), while NU bears the feature of irregular 3D nanocrystals (Figure 1f; Figure S2b, Supporting Information). CuCN shows no detectable Cu nanoparticles, suggesting that the Cu species are highly dispersed at the atomic level, which is consistent with the XRD result (Figure 1a). Aberration‐corrected high‐angle annular dark‐field scanning transmission electron microscopy (AC‐HAADF‐STEM) reveals isolated single atoms, providing direct evidence for the presence of atomically dispersed Cu species (Figure 1e). The presence of both CuCN nanosheets and the NU structure in CuCN/NU indicates their successful intercalation (Figure 1g), which is supported by SEM observations (Figure S2c, Supporting Information). TEM images show that the Def‐CuCN/NU exhibits intimately intercalated interface between CuCN and Def‐NU (Figure 1h). SEM images displays that the morphology of CuCN/NU can be well kept after thermal treatment (Figure S2d, Supporting Information). Energy dispersive X‐ray spectroscopy (EDX) mapping of C, N, O, and Zr in Def‐CuCN/NU reveals the homogeneous distribution of all elements (Figure 1i). The absence of detectable Cu signal is attributed to its ultra‐low loading beyond the detection limit of EDX mapping.

**Figure 2:**
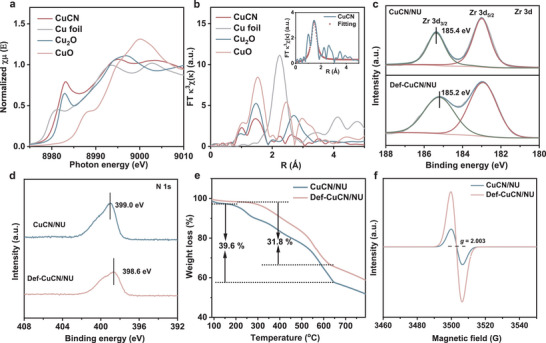
a) Normalized XANES and b) fitted EXAFS results of CuCN and Cu‐related standards at the Cu K‐edge. c) Zr 3d and d) N 1s spectra, e) TGA curves, and f) EPR spectra of CuCN/NU and Def‐CuCN/NU.

The Cu K‐edge X‐ray absorption near‐edge structure (XANES) of CuCN was normalized and analyzed to unveil the valence state and coordination structure of Cu, with CuO, Cu_2_O, and Cu foil as reference standards. As shown in **Figure** **2**a, the absorption edge of CuCN is located between Cu and Cu_2_O, indicating that the valence state of Cu atoms is between 0 and +1. Fourier transform (FT) extended X‐ray absorption fine structure (EXAFS) analysis of CuCN shows a main peak at 1.45 Å without a Cu─Cu peak at ≈2.25 Å, confirming the single‐atomic dispersion of Cu (Figure 2b). XANES fitting indicates a coordination number of 2.4 and a Cu─N bond distance of 1.9 Å, indicating that the Cu atoms are coordinated with two N atoms.^[^ ^46^ ^]^

X‐ray photoelectron spectroscopy (XPS) is employed to characterize the elemental composition and chemical states of the samples. In the Cu 2p spectrum of CuCN (Figure S3, Supporting Information), the peaks at 932.5 and 952.2 eV corresponding to the 2p_3/2_ and 2p_1/2_ levels, respectively, indicate the presence of Cu(I) or Cu(0) species instead of Cu(II).^[^ ^61^ ^]^ These analyses collectively verify that Cu in CuCN is present as isolated single atoms with +1 oxidation state.

The Zr 3d spectrum of CuCN/NU displays 3d_3/2_ and 3d_5/2_ peaks at 185.4 and 183.0 eV, respectively (Figure 2c), characteristic of the Zr^4+^ oxidation state in the Zr_6_O_4_(OH)_4_ secondary building unit.^[^ ^62^ ^]^ In Def‐CuCN/NU, these peaks shift by 0.2 eV toward lower binding energies, indicating increased electron density around the Zr centers.^[^ ^34^ ^]^ The C 1s spectra of CuCN/NU and Def‐CuCN/NU show peaks at 284.8, 285.8, 288.1, and 288.8 eV (Figure S4, Supporting Information), corresponding to C─C/C═C, C─NH_x_, N─C═N, and O─C═O groups, respectively^[^ ^33^, ^63^ ^]^. Thermal treatment induces partial decarboxylation of the MOFs linkers, as evidenced by a significantly reduced COO^−^/Zr ratio derived from the peak areas of Zr 3d_5/2_ and C 1s carboxylate peaks^[^ ^35^ ^]^ (Table S2, Supporting Information). The N 1s spectrum of CuCN/NU can be deconvoluted into four peaks located at 398.9, 399.9, 401.3, and 402.5 eV (Figure S5, Supporting Information), which are assigned to N─Zr coordination bonds, C─N═C groups, N─C_3_ species, and C─N─H structures, respectively.^[^ ^64^, ^65^ ^]^ The formation of N─Zr coordination provides clear evidence of the interfacial linkage between CuCN and NU.^[^ ^66^, ^67^ ^]^ Notably, the N 1s spectrum reveals that the binding energies for Def‐CuCN/NU are lower than those for CuCN/NU (Figure 2d), further corroborating the occurrence of linker decarboxylation in the MOFs upon defect engineering.^[^ ^33^ ^]^ Thermogravimetric analysis (TGA) provides further insights into the structural defects. As shown in Figure 2e, the total weight loss decreases from 39.6% for CuCN/NU to 31.8% for Def‐CuCN/NU, suggesting that the defects mainly arise from the partial removal of organic linkers.^[^ ^33^ ^]^

Electron paramagnetic resonance (EPR) signal is significantly stronger than that of CuCN/NU, indicating that thermal treatment introduces a large number of defect sites in Def‐CuCN/NU, which are associated with the generation of abundant unpaired electrons (Figure 2f). The typical g factor of 2.003 suggests that these unpaired electrons are closely associated with missing‐linker defects at the Zr_6_ nodes. ^[^ ^68^, ^69^ ^]^ These defects are expected to facilitate more efficient separation of photogenerated electron−hole pairs.^[^ ^68^, ^69^ ^]^ Furthermore, the EPR signal of Def‐CuCN/NU under light irradiation in more intense than that in the darkness (Figure S6, Supporting Information), reflecting its effective trapping of photoinduced holes, which is indicative of suppressed charge recombination and enhanced charge separation.

### 2.2 Photocatalytic CH_4_ Oxidation

The photocatalytic oxidation of CH_4_ was conducted in a top‐irradiated high‐pressure autoclave reactor under full‐spectrum Xe lamp irradiation (200 mW cm^−2^) at 25 °C, as illustrated in Figure S7 (Supporting Information). Gaseous products were analyzed by gas chromatography (GC), while liquid‐phase oxygenates including methyl hydroperoxide (CH_3_OOH) and methanol (CH_3_OH) were quantified using ^1^H NMR spectroscopy with a water suppression pulse sequence. The concentration of formaldehyde (HCHO) was determined via the acetylacetone colorimetric method. To verify the origin of the products, control experiments in the absence of CH_4_ or the photocatalyst were performed. The absence of carbon‐containing products under these control conditions confirms that the detected oxygenates are resulted exclusively from photocatalytic oxidation of CH_4_ on the catalyst (Table S3, Supporting Information).

**Figure 3:**
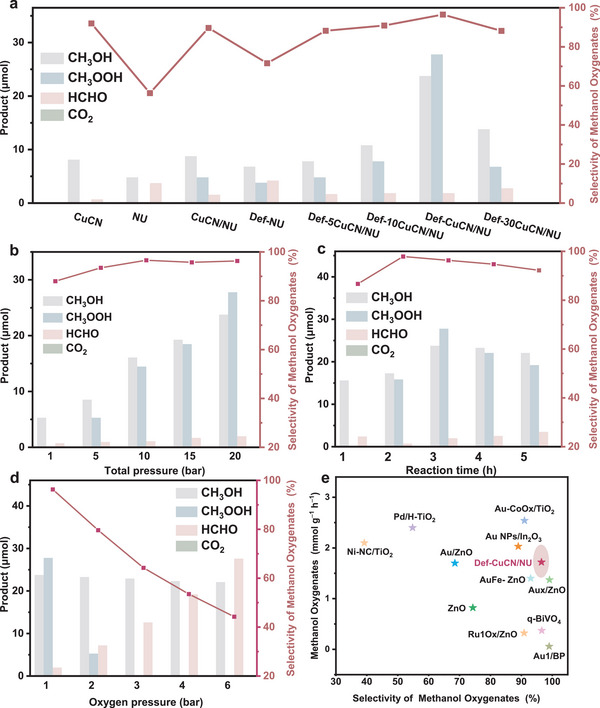
a) Product distributions over the CuCN, NU, CuCN/NU, Def‐NU, Def‐5CuCN/NU, Def‐10CuCN/NU, Def‐CuCN/NU, and Def‐30CuCN/NU catalysts in photocatalytic oxidation of CH_4_ with O_2_. b) Effect of the total pressure on the product distribution over Def‐CuCN/NU in photocatalytic oxidation of CH_4_. The O_2_ pressure was kept at 1 bar. c) Time‐dependent product distribution over Def‐CuCN/NU in photocatalytic oxidation of CH_4_. d) Effect of the CH_4_/O_2_ pressure ratio on the product distribution over Def‐CuCN/NU in photocatalytic oxidation of CH_4_. The total pressure was 20 bar. e) Comparison of the productivity and selectivity of methanol oxygenates over Def‐CuCN/NU with literature results under similar reaction conditions. If unspecified, the reaction conditions are 10 mg of catalyst, 60 mL of water, 19 bar of CH_4_, 1 bar of O_2_, 25°C, 300 < λ < 780 nm, light intensity of ca. 200 mW cm^−2^, stirring rate of 800 rpm, and reaction time of 3 h.

The photocatalytic CH_4_ oxidation activities of various catalysts are shown in **Figure** **3**a. The main oxygenate products detected were CH_3_OH and CH_3_OOH, along with over‐oxidized products such as HCHO and a minor amount of CO_2_ in the gas phase. Herein, CH_3_OH and CH_3_OOH are collectively referred to as methanol oxygenates. Figure 3a shows that pristine CuCN and NU displayed selectivities of 92% and 56% for methanol oxygenates, with yields of 8 and 4.8 µmol, respectively. In contrast, CuCN/NU exhibited significantly enhanced photocatalytic performance, with the yield of methanol oxygenates increased to 13.5 µmol at a high selectivity of 89.7%. The pronounced improvement over NU can be attributed to the formation of a heterojunction that promotes the separation and migration of photogenerated charge carriers, thus enhancing photocatalytic performance.^[^ ^38^, ^40^ ^]^ Moreover, MOFs with structural defects typically feature abundant porous channels and defective sites, which promote the access of reactants and the escape of products, and facilitate charge separation, which is expected to further enhance photocatalytic activity and selectivity.^[^ ^33^, ^34^, ^70^ ^]^ Therefore, CuCN/NU was subjected to thermally treatment to introduce structural defects, yielding a defect‐engineered CuCN/NU catalyst (Def‐CuCN/NU). The catalyst enabled a remarkable increase in the total yield of methanol oxygenates to 51.6 µmol, along with a high selectivity of 96.5%. These results suggest that the introduction of structural defects within MOFs creates a microreactor with confined environment, which not only promotes CH_4_ activation, but also effectively suppresses overoxidation to HCHO and CO_2_. It is also observed that the yield of methanol oxygenates over the Def‐CuCN/NU catalysts exhibited a volcano‐shaped dependence on the loading of CuCN. Specifically, the yields of methanol oxygenates for Def‐NU, Def‐5CuCN/NU, Def‐10CuCN/NU, Def‐CuCN/NU, and Def‐30CuCN/NU catalysts are 10.6, 12.6, 18.6, 51.6, and 20.6 µmol, respectively, with corresponding selectivities of 71.6%, 88.1%, 90.9%, 96.5%, and 88.2%, respectively.

Photocatalytic CH_4_ oxidation based on the Def‐CuCN/NU catalyst was further investigated by systematically varying the total pressure, reaction time, O_2_ pressure, and water volume. The increased system pressure enhances the product yield by improving CH_4_ solubility in the aqueous medium. For instance, the yield of methanol oxygenates is limited to ca. 6 µmol at 1 bar, whereas it reaches a maximum of 51.6 µmol at 20 bar (Figure 3b). The yield of methanol oxygenates follows a volcano‐shaped trend with respect to the reaction time, starting at 15.6 µmol at 1  h, increasing to 51.6 µmol at 3 h, and subsequently declining to 41.3 µmol at 5 h (Figure 3c). The decrease is attributed to time‐dependent overoxidation, during which a portion of the target products is further oxidized to HCHO. The influence of O_2_ pressure was investigated while maintaining a constant total pressure of 20 bar (Figure 3d). A gradual decrease in the selectivity toward methanol oxygenates is observed with the increase in the O_2_ pressure, with the maximum selectivity of 96.5% being achieved at 1 bar, followed by a pronounced drop to 44.3% at 6 bar. CH_3_OOH becomes undetectable while HCHO formation increases markedly at 3 bar and above, suggesting that CH_3_OOH undergoes overoxidation under oxygen‐rich conditions, which deteriorates the overall selectivity. The volume of water has a significant impact on photocatalytic oxidation of CH_4_.^[^ ^43^ ^]^ In the absence of water, CO_2_ is the sole detectable product and in little amount (Figure S8, Supporting Information), highlighting the essential role of water in facilitating CH_4_ activation and inhibiting overoxidation. The selectivity toward methanol oxygenates increases significantly with the increase in the water volume, reaching a maximum of 96.5% at 60 mL, where the yield concurrently reaches 51.6 µmol, corresponding to a production rate of 1718 µmol g^−1^ h^−1^. As compared in Figure 3e, the high yield and excellent selectivity of methanol oxygenates over the Def‐CuCN/NU catalyst surpasses those over most photocatalysts ever reported under similar reaction conditions. Recyclability experiment of the Def‐CuCN/NU catalyst shows that the total amount of the oxidation products declined by 30%, but the selectivity of methanol oxygenates remained constant throughout five consecutive cycles (Figure S9, Supporting Information). After five cycles, the cumulative catalyst mass loss is found to be 38%, so the observed activity drop can be attributed to insufficient recovery of the fine catalyst powders rather than irreversible changes of the physical and chemical properties. The SEM images (Figure S10b, Supporting Information) show that the overall morphology and microstructure of the cycled catalyst remain highly consistent with those of the fresh catalyst (Figure S10a, Supporting Information), with no noticeable aggregation, cracking, or collapse, indicating excellent structural stability under prolonged cycling. The XRD (Figure S10c, Supporting Information) and IR (Figure S10d, Supporting Information) analyses further confirm the preservation of the crystal structure and chemical framework after cycling. Meanwhile, XPS analysis reveals that the chemical states and surface composition of Zr (Figure S10e, Supporting Information) and N (Figure S10f, Supporting Information) in the cycled catalyst remain essentially unchanged, demonstrating that the active sites possess robust chemical stability, further confirming the excellent stability of the catalyst.

### 2.3 Optical and Electrochemical Properties

**Figure 4:**
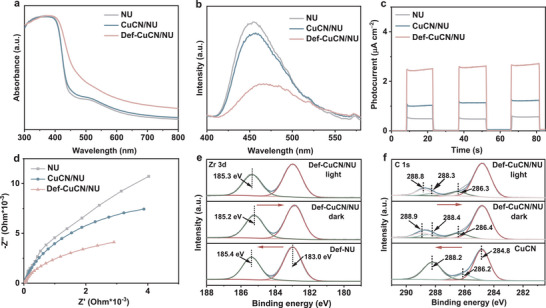
a) UV‐vis DRS, b) PL spectra, c) transient photocurrent responses, and d) EIS Nyquist plots of NU, CuCN/NU, and Def‐CuCN/NU. High‐resolution XPS spectra of Def‐CuCN/NU under dark and light irradiation: e) Zr 3d and f) C 1s.

In UV‐visible diffuse reflectance spectra (UV‐vis DRS) (**Figure** **4**a), the absorption edges of CuCN/NU and NU show no significant difference, indicating that the formation of the heterojunction does not alter the bandgap of NU. Notably, Def‐CuCN/NU exhibits a broader visible light absorption range, showing that defect formation during thermal treatment narrows down the bandgap, which enhances visible‐light absorption and improves photocatalytic performance.

Steady‐state photoluminescence (PL) spectra (Figure 4b) show that the intensity of CuCN/NU is lower than that of NU, indicating that the formation of heterojunction suppresses of exciton recombination and prolongs the lifetime of photogenerated excitons. The further reduction in PL intensity for Def‐CuCN/NU indicates that defect engineering effectively prolongs the lifetime of the excitons. Transient PL decay spectra (Figure S11, Supporting Information) show that Def‐CuCN/NU exhibits a shorter exciton lifetime than CuCN/NU, demonstrating that the defects accelerate the separation of photogenerated electron−hole pairs at the heterojunction. Photoelectrochemical current response curves reveal that CuCN/NU exhibits a stronger photocurrent response than NU, confirming that the heterojunction promotes the separation and transport of the charge carriers. It is worth noting that Def‐CuCN/NU exhibits an even markedly enhanced photocurrent response (Figure 4c), indicative of significantly improved charge carrier separation and transport. Electrochemical impedance spectroscopy (EIS) Nyquist plots (Figure 4d) demonstrate that Def‐CuCN/NU displays a smaller semi‐circular arc radius than both NU and CuCN/NU, indicating its lower electron transport resistance. The synergistic effect of enhanced visible‐light absorption and more efficient charge carrier separation and transport can contribute to the improved photocatalytic performance of Def‐CuCN/NU.

Considering that Def‐CuCN/NU is composed of CuCN and Def‐NU components, XPS valence band(*E*_VB_) analysis was performed to its band structure (Figure S12a,b, Supporting Information). The results show that the valence band edges are 2.27 eV for CuCN and 3.12 eV for Def‐NU. Furthermore, UV−vis diffuse reflectance spectroscopy and Tauc plots (Figure S12c,d, Supporting Information) were employed to determine the optical bandgaps(*E*_g_) based on the Kubelka−Munk function, which were 2.67 and 2.84 eV, respectively. Based on the relation *E*_g_ *= |E*_CB_ *− E*_VB_ *|*, the conduction band(*E*_CB_) positions of CuCN and Def‐NU were estimated to be −0.40 and 0.28 V, respectively. The band structures demonstrate that CuCN and Def‐NU form a heterojunction (Figure S12e, Supporting Information).

In situ XPS was conducted to track the migration of photogenerated electrons under light irradiation, thereby clarifying the charge transfer pathway in Def‐CuCN/NU.^[^ ^70^ ^]^ The Zr 3d spectrum of Def‐NU exhibits characteristic 3d_3/2_ and 3d_5/2_ peaks at 185.4 and 183.0 eV (Figure 4e). After CuCN was incorporated into the Def‐NU microreactor, the Zr 3d peaks shifted negatively by ≈0.1 eV, indicating an increased electron density in Def‐NU due to interfacial electron transfer from CuCN. Under illumination, however, the Zr 3d peaks shifted positively by ≈0.2 eV, suggesting a back‐transfer of electrons from Def‐NU to CuCN.^[^ ^71^ ^]^ For CuCN, the C 1s spectrum can be deconvoluted into three peaks at 284.8, 286.2, and 288.2 eV, corresponding to C─C/C═C, C─NH_x_, and N─C═C species,^[^ ^70^ ^]^ respectively (Figure 4f). In Def‐CuCN/NU, a new peak emerges at 288.9 eV, assigned to the O─C═O bond of the MOFs framework.^[^ ^71^ ^]^ In contrast to the Zr 3d trend, the C 1s binding energies show a positive shift of ≈0.2 eV upon contact, followed by a negative shift of ≈0.1 eV under light irradiation. Taken together, the opposite electron transfer behaviors of Def‐NU and CuCN during interfacial contact and photoexcitation provide strong evidence that the heterojunction follows an S‐scheme charge transfer mechanism.^[^ ^72^, ^73^, ^74^, ^75^ ^]^

### 2.4 Reaction Mechanism of CH_4_ Photocatalytic Oxidation

**Figure 5:**
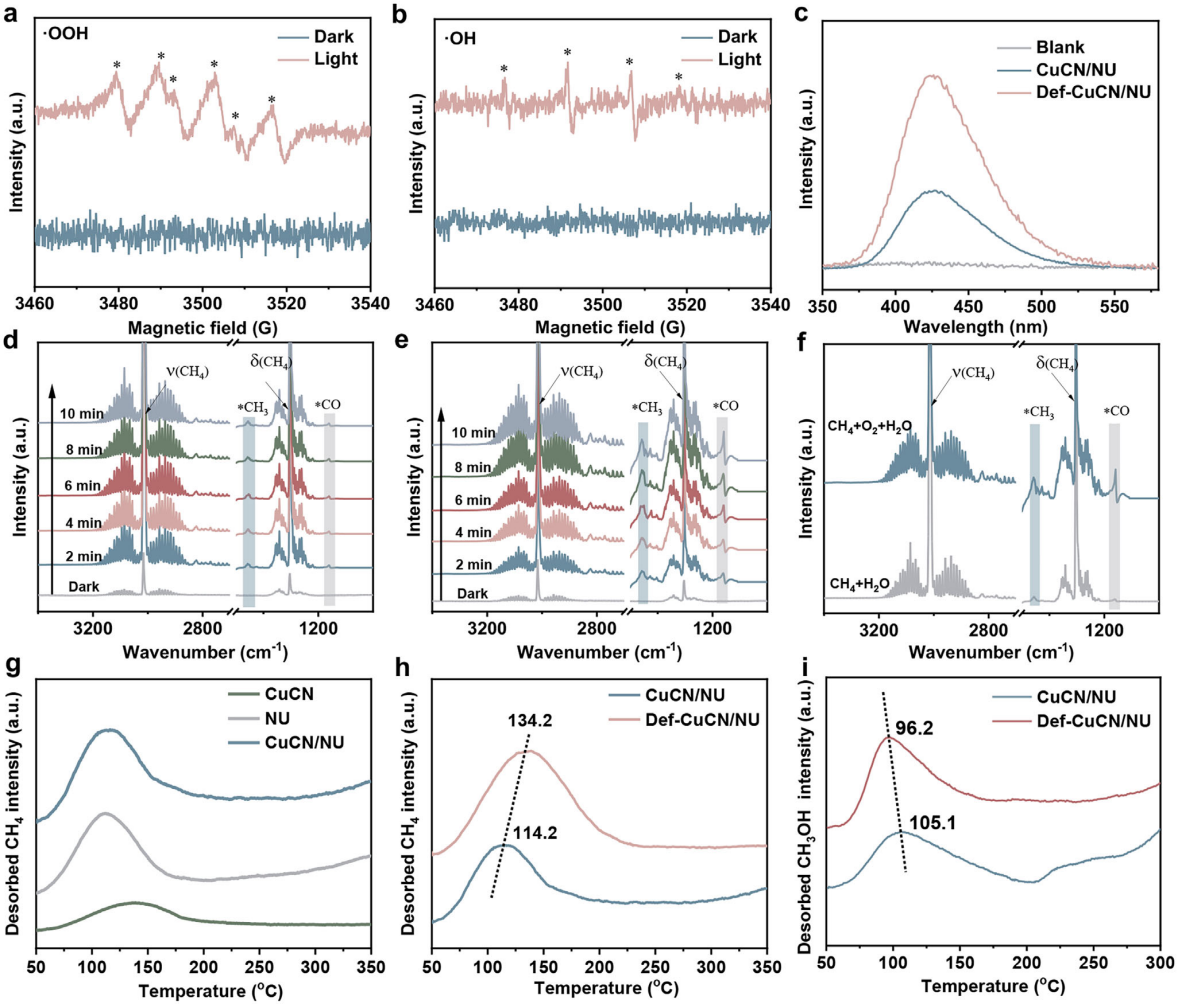
a,b) In situ EPR spectra of Def‐CuCN/NU dispersed in CH_3_OH and H_2_O under O_2_, using DMPO as a spin‐trapping agent to detect ·OOH and ·OH radicals, respectively. c) Fluorescence spectra of CuCN/NU and Def‐CuCN/NU for ·OH detection using terephthalic acid as a fluorescent probe. d) In situ DRIFTS spectra of Def‐CuCN/NU under light irradiation in the presence of humidified CH_4_. e) In situ DRIFTS spectra of Def‐CuCN/NU under light irradiation in the presence of humidified CH_4_/O_2_. f) Comparative in situ DRIFTS spectra of Def‐CuCN/NU after 10 min exposure to humidified CH_4_ and humidified CH_4_/O_2_ under light irradiation. g) CH_4_‐TPD profiles of CuCN, NU, and CuCN/NU. h) CH_4_‐TPD profiles of CuCN/NU and Def‐CuCN/NU. i) CH_3_OH‐TPD profiles of CuCN/NU and Def‐CuCN/NU.

In the photocatalytic oxidation of CH_4_,·OOH and ·OH are identified as the primary reactive oxygen species (ROS) when molecular O_2_ is employed as the oxidant.^[^ ^24^, ^76^ ^]^ The formation of ROS during photocatalysis is confirmed by in situ EPR spectroscopy using 5,5‐dimethyl‐1‐pyrroline N‐oxide (DMPO) as a spin‐trapping agent. The generation of ·OOH and ·OH radicals was confirmed under light irradiation in methanol and water solutions, respectively, as evidenced by the characteristic EPR signals of their corresponding DMPO adducts (**Figure** **5**a,b). It is accepted that the oxidation of CH_4_ to C1 oxygenates follows a stepwise reaction sequence: CH_4_ → CH_3_OOH → CH_3_OH → HCHO.^[^ ^77^ ^]^ Among these steps, the activation of CH_4_ to form methyl radical (·CH_3_) is the rate‐determining step, which can proceed via two primary pathways: hydrogen abstraction mediated by photogenerated holes (h^+^) (Equation (1))^[^ ^78^ ^]^ or hydroxyl radicals (·OH) (Equation (2))^[^ ^79^ ^]^:

(1) $${\mathrm{CH}}_{4}+h^{+}\to \cdot {\mathrm{CH}}_{3}+H^{+}$$

(2) $$CH_{4}+\cdot \mathrm{OH}\to \cdot CH_{3}+H_{2}O$$

On the Def‐CuCN/NU catalyst, the methyl radical (·CH_3_) is proposed to be generated via hydrogen atom abstraction from CH_4_ by ·OH radicals, as described in Equation (2). This is corroborated by the complete suppression of the formation of methanol oxygenates upon scavenging ∙OH radicals (Figure S13, Supporting Information). The activated ·CH_3_ radicals can undergo further transformation via two distinct pathways. In one pathway, they react with ·OOH to yield a series of reactive intermediates and products (Equations (3−5)).^[^ ^77^, ^80^ ^]^ Alternatively, ·CH_3_ can directly couple with ·OH to form CH_3_OH (Equation (6)).^[^ ^81^ ^]^

(3) $$\;CH_{3}+\cdot \mathrm{OOH}\to CH_{3}\mathrm{OOH}$$

(4) $$CH_{3}\mathrm{OOH}\;+2H^{+}+2e^{-}\to CH_{3}\mathrm{OH}+H_{2}O$$

(5) $$CH_{3}\mathrm{OOH}\to \mathrm{HCHO}\;+H_{2}O$$

(6) $$CH_{3}+\cdot \mathrm{OH}\to CH_{3}\mathrm{OH}$$

In the absence of ·OOH radicals, Equations (3−5) are significantly suppressed (Figure S11, Supporting Information). Nevertheless, trace amounts of CH_3_OH were still detected, with a yield about one order of magnitude lower than that observed under O_2_ atmosphere. This suggests that Equation(6) may proceed as a secondary pathway, further confirming the involvement of ·OH radicals in the reaction process. The presence of·OH radicals was further verified using terephthalic acid as a fluorescence probe under light irradiation (Figure 5c). Notably, the fluorescence intensity observed for Def‐CuCN/NU was significantly higher than that for CuCN/NU, indicating enhanced generation of ·OH radicals. The higher concentration of ·OH radicals facilitates CH_4_ activation and hence promotes the formation of methanol oxygenates. The ·OH radicals can be generated via two primary routes: H_2_O oxidation (Equation(7))^[^ ^77^, ^80^ ^]^ or H_2_O_2_ reduction (Equation (8))^[^ ^77^, ^80^ ^]^:

(7) $$H_{2}O+h^{+}\to \cdot \mathrm{OH}+H^{+}$$

(8) $$H_{2}O_{2}+e^{-}\to \cdot \mathrm{OH}+OH^{-}$$

It should be noted that no H_2_O_2_ was detected after 1 h of light irradiation over Def‐CuCN/NU in a reaction system containing only O_2_. This indicates that Equation(7) is the more probable pathway for ·OH radical generation. In other words, ·OH radicals are mainly generated via the oxidation of H_2_O, rather than through the reduction of O_2_ to H_2_O_2_ followed by subsequent reduction. Meanwhile, ·OOH radicals can be generated via two possible pathways: the proton‐coupled O_2_ reduction (Equation(9))^[^ ^80^ ^]^ or the oxidation of H_2_O_2_ (Equation(10)^[^ ^82^ ^]^:

(9) $$O_{2}+e^{-}+H^{+}\to \cdot \mathrm{OOH}$$

(10) $$H_{2}O_{2}+h^{+}\to \cdot \mathrm{OOH}\;+H^{+}$$

Although both O_2_ reduction and H_2_O_2_ oxidation are thermodynamically capable of generating ·OOH radicals, the absence of detectable H_2_O_2_ in the reaction system suggests that ·OOH radicals are primarily produced via the reduction of O_2_ on Def‐CuCN/NU. In addition, as a single‐electron transfer process, proton‐coupled O_2_ reduction (Equation(9)) is kinetically highly favorable.^[^ ^83^, ^84^ ^]^

To gain insights into the adsorption states of reactants and the evolution of reaction intermediates during photocatalysis, in situ diffuse reflectance infrared Fourier transform spectroscopy (DRIFTS) characterization was conducted. Upon exposure of the Def‐CuCN/NU catalyst to humidified CH_4_ or CH_4_/O_2_ atmosphere, two prominent absorption bands were observed at 3015 and 1303 cm^−1^ (Figure 5d,e), which are characteristic of adsorbed methane molecules.^[^ ^45^ ^]^ Upon light irradiation, the distinct absorption band at ≈1471 cm^−1^ (Figure 5d,e), assigned to the bending mode of ∙CH_3_ species, provides clear evidence for the rapid activation of CH_4_ on the catalyst.^[^ ^10^ ^]^ Concurrently, the appearance and gradual intensification of the band at ≈1091 cm^−1^ (Figure 5d), attributed to the C─O stretching vibration of CH_3_OH,^[^ ^10^ ^]^ further confirms the efficient coupling between ∙CH_3_ and ∙OH radicals under light irradiation.^[^ ^10^ ^]^ However, under the reaction atmosphere of humidified CH_4_, the bands at 1471 and 1091 cm^−1^ showed no appreciable increase in intensity over time under light irradiation (Figure 5e), and their overall intensities were substantially lower than those under the humidified CH_4_/O_2_ atmosphere at the same time (Figure 5f). The diminished signal intensity further underlines the indispensable role of O_2_ in promoting the photocatalytic oxidation of CH_4_ to CH_3_OH oxygenates, which is consistent with the reaction mechanism proposed above. Moreover, temperature‐programmed desorption of CH_4_ (CH_4_‐TPD) measurements reveal that NU exhibits a significantly stronger CH_4_ adsorption peak than CuCN (Figure 5g), indicating that CH_4_ is primarily adsorbed within NU. Furthermore, Def‐CuCN/NU exhibited larger CH_4_ desorption peak area and higher desorption temperature as compared to CuCN/NU (Figure 5h), indicating that introducing defects to NU creates more accessible and stronger adsorption sites for CH_4_. On the other hand, CH_3_OH‐TPD measurements evidenced the lower desorption temperature of CH_3_OH on Def‐CuCN/NU than on CuCN/NU (Figure 5i), which shortens its contact with ·OH radicals, thus avoiding overoxidation to HCHO and CO_2_. Therefore, Def‐CuCN/NU with abundant pore channels functions as a microreactor, which not only facilitates the rapid coupling of ·OOH and ·CH_3_ radicals within the confined space, but also strengthens the adsorption of CH_4_ while weakens the desorption of CH_3_OH, thus boosting the overall activity and selectivity in photocatalytic oxidation of CH_4_ to methanol oxygenates.

**Figure 6:**
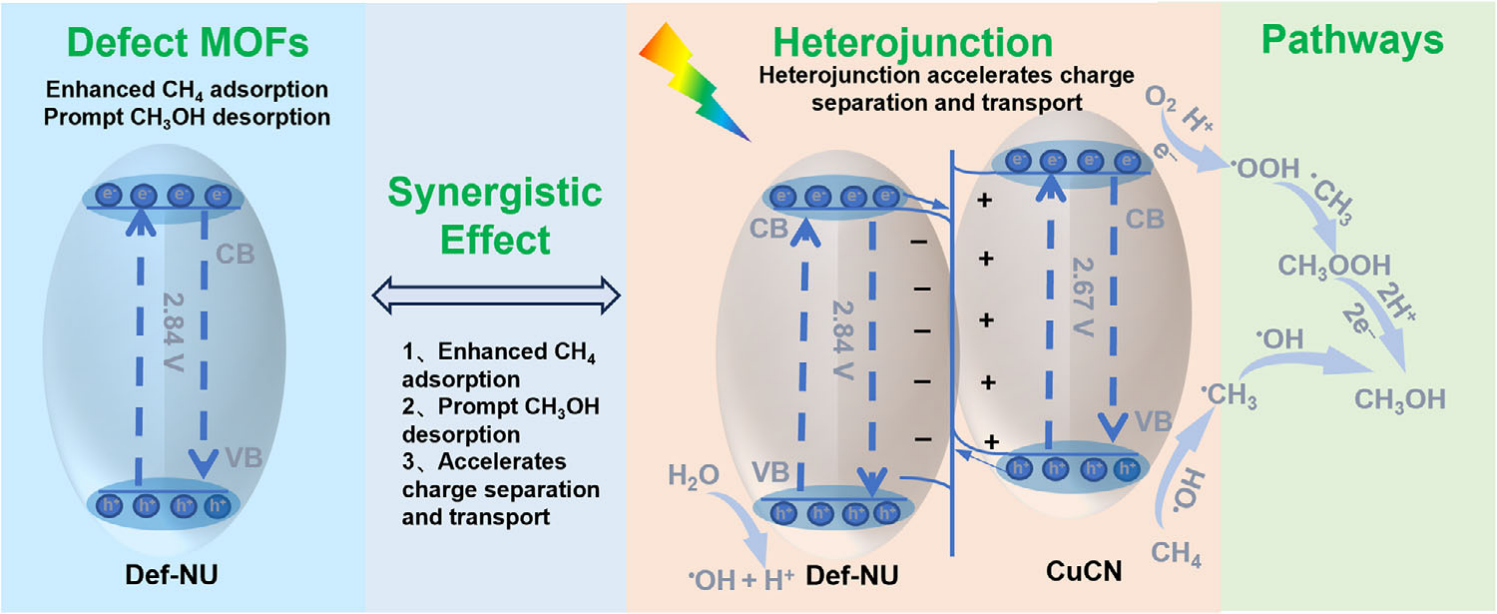
Proposed reaction mechanism for the photocatalytic oxidation of CH_4_ to methanol oxygenates over Def‐CuCN/NU.

In light of the experimental observations and mechanistic insights, we propose a plausible reaction pathway for photocatalytic CH_4_ oxidation over the Def‐CuCN/NU heterojunction catalyst. As illustrated in **Figure** **6**, the catalyst consists of CuCN and Def‐NU, where Def‐NU acts as a microreactor to enhance CH_4_ adsorption and facilitate CH_3_OH desorption. Together, CuCN and Def‐NU construct an S‐scheme heterojunction that enables efficient charge separation and transfer while maintaining strong redox potentials. Under light irradiation, photogenerated holes on Def‐NU oxidize H_2_O to yield ∙OH radicals, whereas electrons on CuCN reduce O_2_ to ·OOH species. The rate‐determining step involves ∙OH radicals abstracting hydrogen atoms from CH_4_ to generate ∙CH_3_ radicals, which predominantly react with ·OOH to form CH_3_OOH, subsequently converting into CH_3_OH. As a side reaction, the ∙CH_3_ radicals combine with ∙OH to produce CH_3_OH. Meanwhile, acting as a microreactor, Def‐CuCN/NU promotes the rapid desorption of CH_3_OH, thereby minimizing its prolonged exposure to highly oxidative ∙OH radicals and effectively suppressing overoxidation to HCHO and CO_2_.

## 3 Conclusion

In summary, we have developed defect‐engineered metal–organic framework (MOF)‐based microreactors by interclacating polymeric carbon nitride (CN) anchored with copper single atoms (Cu SA) into a defective NH_2_‐UiO‐66 framework (Def‐NU). The Def‐CuCN/NU catalyst demonstrates exceptional performance for the selective photocatalytic oxidation of CH_4_ to methanol oxygenates, achieving a high production rate of 1718 µmol g^−1^ h^−1^ and a selectivity of 96.5%. Mechanistic studies reveal that the type‐S heterojunction formed between CuCN and Def‐NU markedly enhances charge carrier separation and transport to facilitate efficient activation of H_2_O, O_2_, and CH_4_. Concurrently, defect engineering generates abundant pore structures that significantly improve CH_4_ adsorption and enable rapid CH_3_OH desorption. This work not only offers a novel approach for the highly selective conversion of methane to value‐added liquid products, but also provides theoretical insights and practical guidance for designing photocatalysts with tailored product selectivity.

The authors have cited additional references within the Supporting Information.

## Conflict of Interest

The authors declare no conflict of interest.

## Supporting information



Supporting Information

## Data Availability

The data that support the findings of this study are available from the corresponding author upon reasonable request.
