# Supplementary material for: Defective MOFs Microreactor with Heterojunction for Selective Methane Photocatalysis via Desorption‐Driven Overoxidation Suppression
Source: Adv Sci (Weinh). 2025 Oct 27;13(3):e13507. doi: 10.1002/advs.202513507 (PMC12806371; doi:10.1002/advs.202513507)
Supplement: Supplementary file 1 — Supporting Information [file ADVS-13-e13507-s001.docx]

Supporting Information

**Defective MOFs Microreactor with Heterojunction for Selective Methane Photocatalysis via Desorption-Driven Overoxidation Suppression**

Bo Feng, Danning Feng, Kun Wan, Yan Pei, Baoning Zong*, Hexing Li, Wei Li*, Minghua Qiao*

**Experimental Section**

**Materials**

Melamine, methanol (CH_3_OH), copper(II) chloride (CuCl_2_), hydrogen peroxide (H_2_O_2_, 30 wt%), Hydrochloric acid (HCl, 36–38%), Sodium hydroxide (NaOH), and 5,5-dimethyl-1-pyrroline N-oxide (DMPO) were purchased from Sinopharm Chemical Reagent Co. Ammonium acetate (CH_3_COONH_4_), acetylacetone, 2-aminoterephthalic acid (C_8_H_7_NO_4_) (NH_2_-BDC), N,N-dimethylformamide (DMF, C_3_H_7_NO), zirconium tetrachloride (ZrCl_4_), Para-benzoquinone (C_6_H_4_O_2_), salicylic acid (C_7_H_6_O_3_) and terephthalic acid (C_8_H_6_O_4_) were obtained from Aladdin Chemical Technology Co. All reagents were of analytical grade and used without further purification. 5 wt% Nafion solution was purchased from Sigma-Aldrich. Ultra-high purity (99.99%) CH_4_ and He gases were purchased from Air Liquide, while N_2_ and O_2_ gas of the same purity was obtained from Shanghai Domoai Purification Gas Co., Ltd.

**Catalyst preparation**

**Synthesis of C_3_N_4_ (CN)**

Melamine (3.0 g) was placed in a crucible and calcined at 550 °C at 5 °C min^−1^ in N_2_ flow for 4 h. The obtained product is referred to as CN.

**Synthesis of Cu SA Modified C_3_N_4_ (CuCN)**

CuCN was synthesized by stirring 3.0 g of melamine in 40 mL of deionized water at 60 °C for 30 min, followed by the dropwise addition of 2 mL of 0.5 M CuCl_2_ solution. The resulting suspension was dried and then calcined at 550 °C for 4 h under N_2_ atmosphere. The Cu loading was determined to be 0.68 wt% by ICP−OES analysis.

**Synthesis of CuCN/NH_2_-UiO-66-CuCN/NU (CuCN/NU)**

CuCN (x wt%) was dispersed in 40 mL of DMF and stirred until homogeneity. ZrCl_4_ (0.82 g) and NH_2_-BDC (0.58 g) were added, followed by dropwise addition of 0.4 mL HCl under continuous stirring. The mixture was transferred to a 100 mL Teflon-lined autoclave and heated at 120 °C for 24 h. After being cooled down to room temperature, the solid was collected by centrifugation, washed sequentially with DMF, methanol, and water three times each, and dried at 80 °C for 24 h. The product was denoted as xCuCN/NU, where *x* represents the CuCN content relative to NU (*x* = 0, 5, 10, 20, 30 wt%). Notably, 0CuCN/NU corresponds to pure NU, and CuCN/NU refers to 20CuCN/NU.

**Synthesis of Defective CuCN/****NH_2_-UiO-66 (Def-CuCN/NU)**

The as-prepared *x*CuCN/NU powders were dried in vacuo at 200 °C for 6 h to generate structural defects, yielding defective *x*CuCN/NU (Def-*x*CuCN/NU).

**Photocatalytic reduction of O_2_ to H_2_O_2_**

Photocatalytic H_2_O_2_ synthesis was performed under conditions analogous to CH_4_ oxidation and quantified using the DPD–POD colorimetric method^[43]^. Typically, 10 mg catalyst were dispersed in 60 mL deionized water, followed by sealing the reactor and purging it with O_2_ for 20 min. Then, the reactor was irradiated with full-spectrum Xe lamp light (200 mW cm^-2^) while maintaining the reaction temperature at 25 °C using a circulating cooling water bath.

**Photocatalytic oxidation of CH_4_**

Photocatalytic oxidation of CH_4_ was performed in a top-irradiation high-pressure reactor (Shanghai Baikal Technology, BKGH). 10 mg catalyst suspended in 60 mL deionized water were purged with O_2_ for 20 min, pressurized with 1 bar O_2_ and 19 bar CH_4_, followed by full-spectrum Xe lamp irradiation (200 mW cm^-2^) at 25 °C using a circulating cooling water bath.

The gaseous products were analyzed by gas chromatography equipped with a TDX-1 column and flame ionization detector. Liquid-phase methanol and methyl hydroperoxide were quantified via ^1^H NMR spectroscopy with DMSO as an internal standard. HCHO was quantified by combining 0.5 mL of the liquid sample with 2.0 mL of a reagent solution—prepared by dissolving 15 g ammonium acetate, 0.3 mL acetic acid, and 0.2 mL pentane-2,4-dione in 100 mL water—and measuring the absorbance at 413 nm using a UV–vis spectrophotometer (UV-3600 Plus, Shimadzu).

**Statistical Analysis**

All experimental data were processed and analyzed using standard statistical methods. Prior to analysis, raw data were evaluated for outliers and, when necessary, normalized to account for systematic variations. Data are presented as mean ± standard deviation (SD), with deviations within 5–8%, based on at least three independent experiments (n ≥ 3).

**Catalyst characterization**

Fourier-transform infrared (FTIR) spectra were collected on a ThermoFisher Nicolet iS10 spectrometer using the standard KBr pellet technique. X-ray diffraction (XRD) patterns were obtained on a Bruker AXS D8 Advance diffractometer using Cu Kα radiation (*λ* = 1.5406 Å, 40 kV, 40 mA). The specific surface area and pore volume were determined using the Brunauer−Emmett−Teller (BET) method. For CuCN, nitrogen adsorption–desorption measurements were performed on a Micromeritics Tristar 3020 analyzer, while the MOFs-based samples were analyzed using a Micromeritics ASAP 2020 instrument. Surface morphology was examined by scanning electron microscopy (SEM) using a ZEISS Gemini 300 system. Microstructural imaging was conducted by transmission electron microscopy (TEM) on an FEI Tecnai G2S-Twin F20 instrument equipped with an Oxford X-Max 80T EDS detector. Elemental composition and chemical states were analyzed via X-ray photoelectron spectroscopy (XPS) using a Thermo ESCALAB 250Xi system with monochromated Al Kα radiation. UV−vis diffuse reflectance spectra (DRS) were recorded on a Perkin−Elmer Lambda 650S spectrophotometer. Steady-state photoluminescence (PL) spectra were collected using a Varian Cary Eclipse fluorometer with 375 nm laser excitation. Time-resolved PL measurements were performed on an Edinburgh Instruments FLS1000 system employing a Xenon lamp (*λ* = 375 nm) as the excitation source.

EPR spectra were recorded at room temperature using a Bruker EMX X-band spectrometer (9.40 GHz). For in situ radical detection, 5 mg catalyst were suspended in 2 mL methanol or water to detect ·O_2_^−^ or ·OH radicals, respectively, with the addition of 20 μL DMPO. After Xenon lamp irradiation, the mixture was analyzed with a Bruker EMX X-band spectrometer.

The formation of hydroxyl radicals (∙OH) was detected using terephthalic acid as a probe. A 25 mL alkaline solution of terephthalic acid (0.5 mmol L^−1^ terephthalic acid in 2 mmol L^−1^ NaOH) was prepared, into which 20 mg of the photocatalyst was introduced and uniformly dispersed. Following 1 h of exposure to full-spectrum irradiation, the characteristic emission of 2-hydroxyterephthalic acid was measured at 315 nm using a Hitachi F-4600 fluorescence spectrometer.

Radical trapping experiments were conducted to investigate the mechanism of photocatalytic CH_4_ oxidation. Para-benzoquinone (0.2 mmol) and salicylic acid (0.2 mmol) were employed as scavengers for ∙O_2_^−^ and ∙OH radicals, respectively. All other reaction conditions were identical with those used in the photocatalytic CH_4_ oxidation.

CH_4_-TPD was performed using a Micromeritics ChemiSorb 2750 instrument. 50 mg catalyst were heated to 100 °C at a rate of 10 °C min^−1^ under He atmosphere and held for 1 h. After being cooled down to 50 °C, the catalyst was saturated with CH_4_ (50 cm^3^ min^−1^) for 1 h, followed by isothermal removal of physically adsorbed CH_4_ in He for 30 min. The desorption of CH_4_ was monitored by a thermal conductivity detector at a heating rate of 10 °C min^−1^ to 350 °C in the He atmosphere. CH_3_OH-TPD procedure is similar to that of CH_4_-TPD, except that CH_3_OH is introduced by bubbling He through methanol for 10 min.

Photoelectrochemical behavior was examined by employing a conventional three-electrode cell system on a CHI760E (Shanghai, Chenhua) electrochemical workstation. A platinum sheet and an Ag/AgCl electrode served as the counter and reference electrodes, respectively. The working electrode was fabricated by depositing a catalyst ink—prepared from 5 mg catalyst, 1 mL ethanol, and 25 μL 5 wt% Nafion—onto a 1 × 2 cm^2^ FTO substrate, followed by drying at 60 °C for 2 h. Electrochemical impedance spectroscopy and transient photocurrent measurements were performed in 0.1 M Na_2_SO_4_ under simulated solar irradiation at 0.2 V vs Ag/AgCl. Mott–Schottky plots were recorded under identical conditions with a 10 mV AC amplitude at 2 kHz.

DRIFTS measurements were conducted on a Nicolet 6700 with an in situ accessory. The catalyst was pretreated in N_2_ atmosphere at 200 °C for 1 h, then cooled to room temperature. CH_4_ or CH_4_/O_2_ gases was introduced through a glass tube containing water. The full-spectrum light irradiation was directed through the DRIFTS window, and the data were collected every 2 min, with 0 min indicating gas introduction prior to light irradiation.


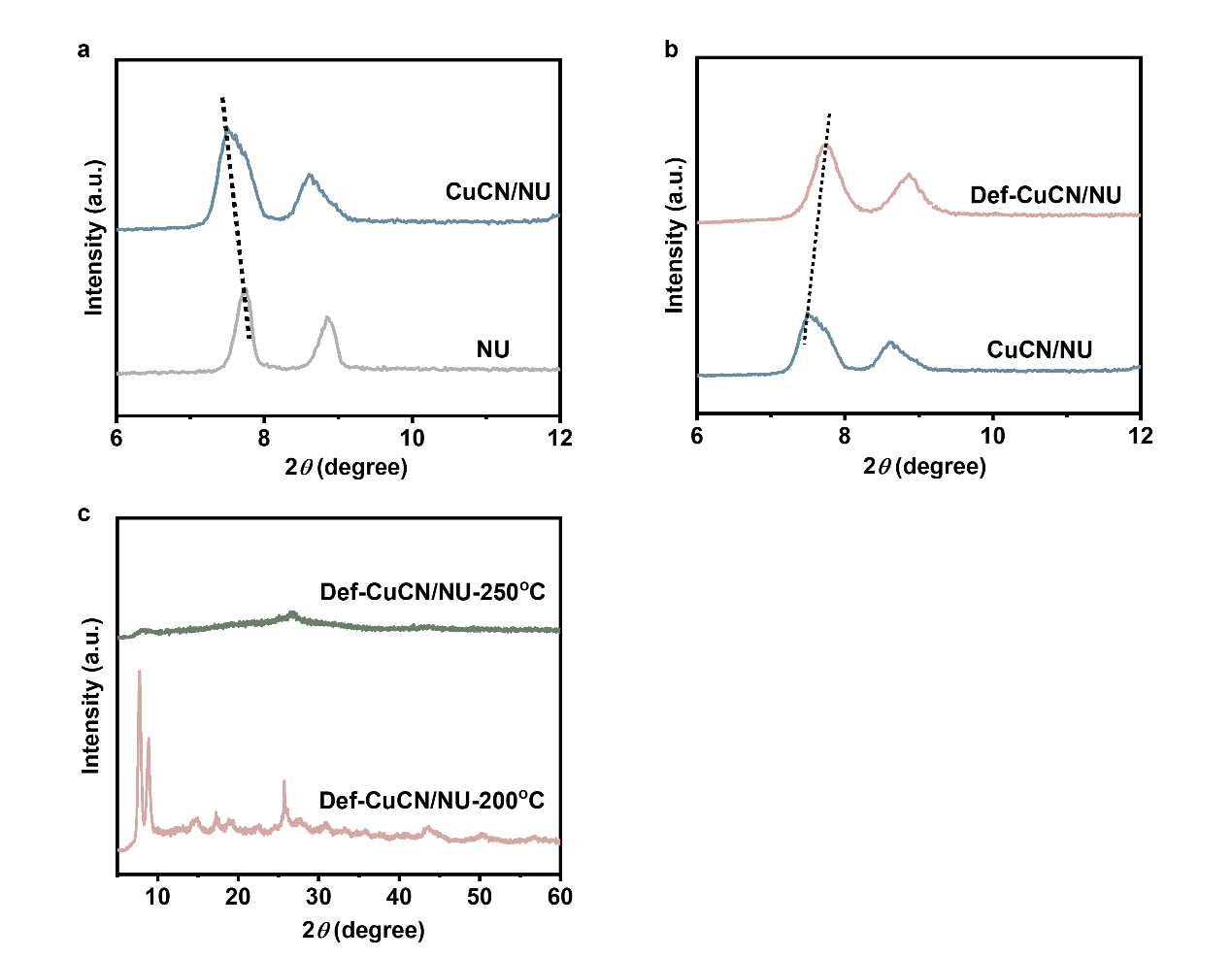


**Figure S1.** Magnified XRD patterns of (a) NU and CuCN/NU and (b) CuCN/NU and Def-CuCN/NU. (c) XRD patterns of Def-CuCN/NU annealed at different temperatures.


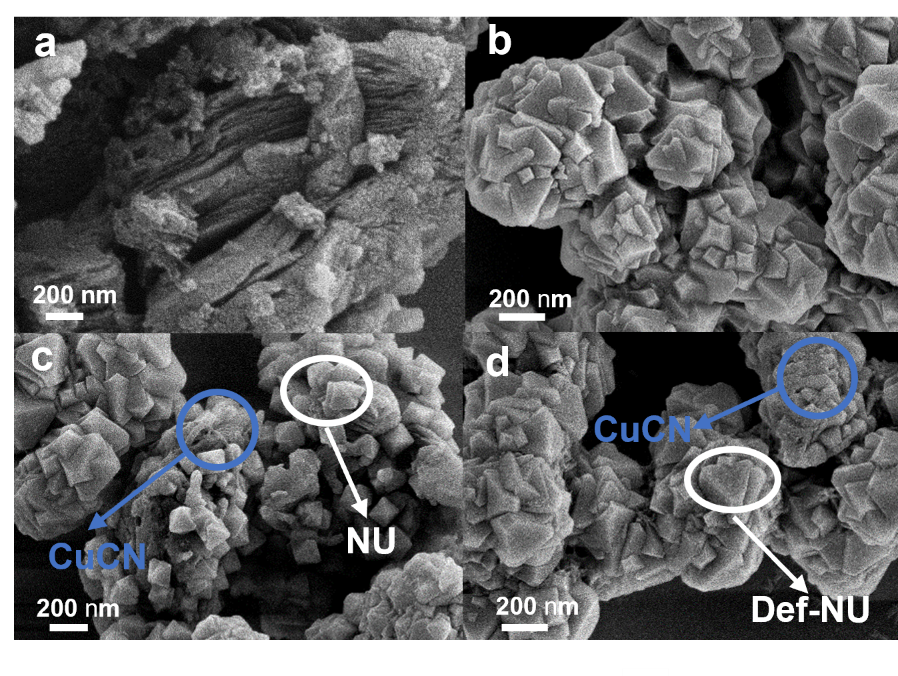


**Figure S2.** SEM images of (a) CuCN, (b) NU, (c) CuCN/NU, and (d) Def-CuCN/NU.

**Figure S3.** Cu 2p spectrum of CuCN.

**Figure S4.** XPS spectra of CuCN/NU and Def-CuCN/NU in the C 1s region.

**Figure S5.** XPS spectrum of CuCN/NU in the N 1s region.

**Figure S6.** EPR spectra of Def-CuCN/NU in the dark and light.


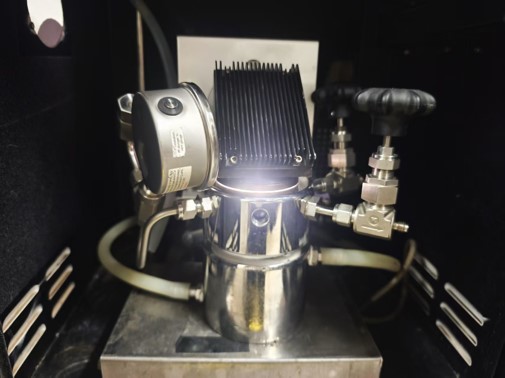


**Figure S7.** Photograph of the photocatalytic CH_4_ oxidation setup.


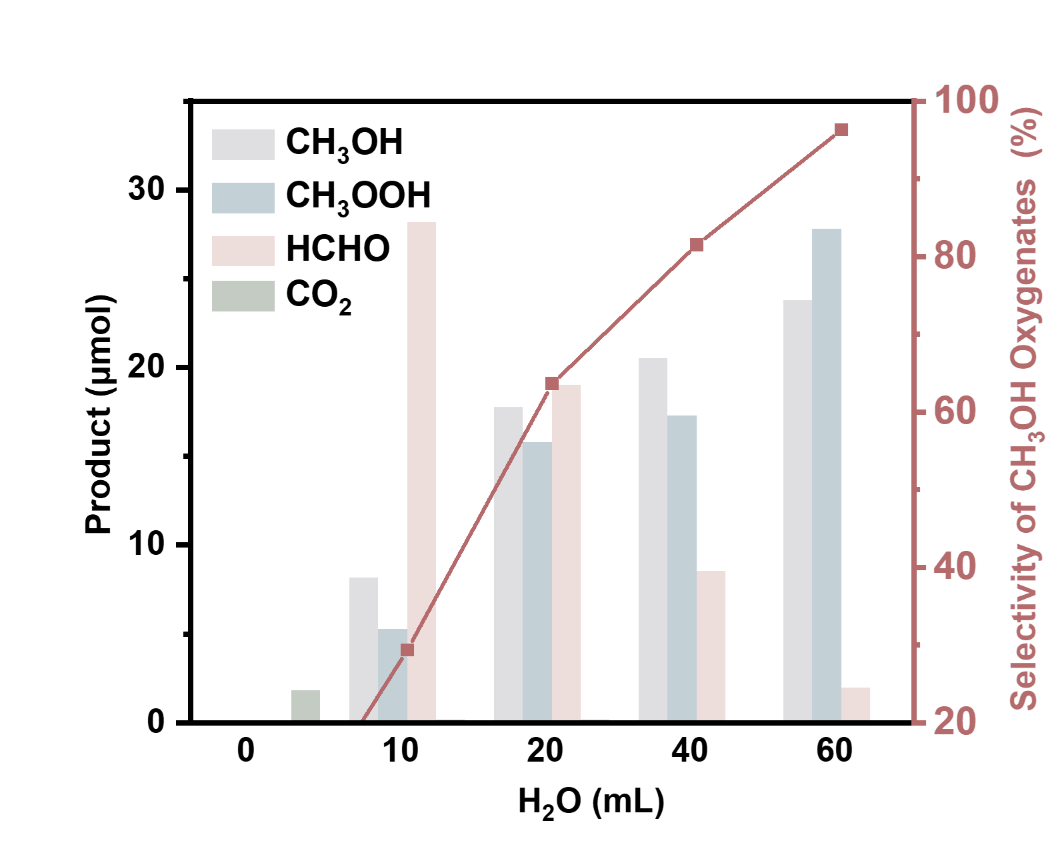


**Figure S8.** Effect of the volume of water on the product distribution over Def-CuCN/NU in photocatalytic oxidation of CH_4_.


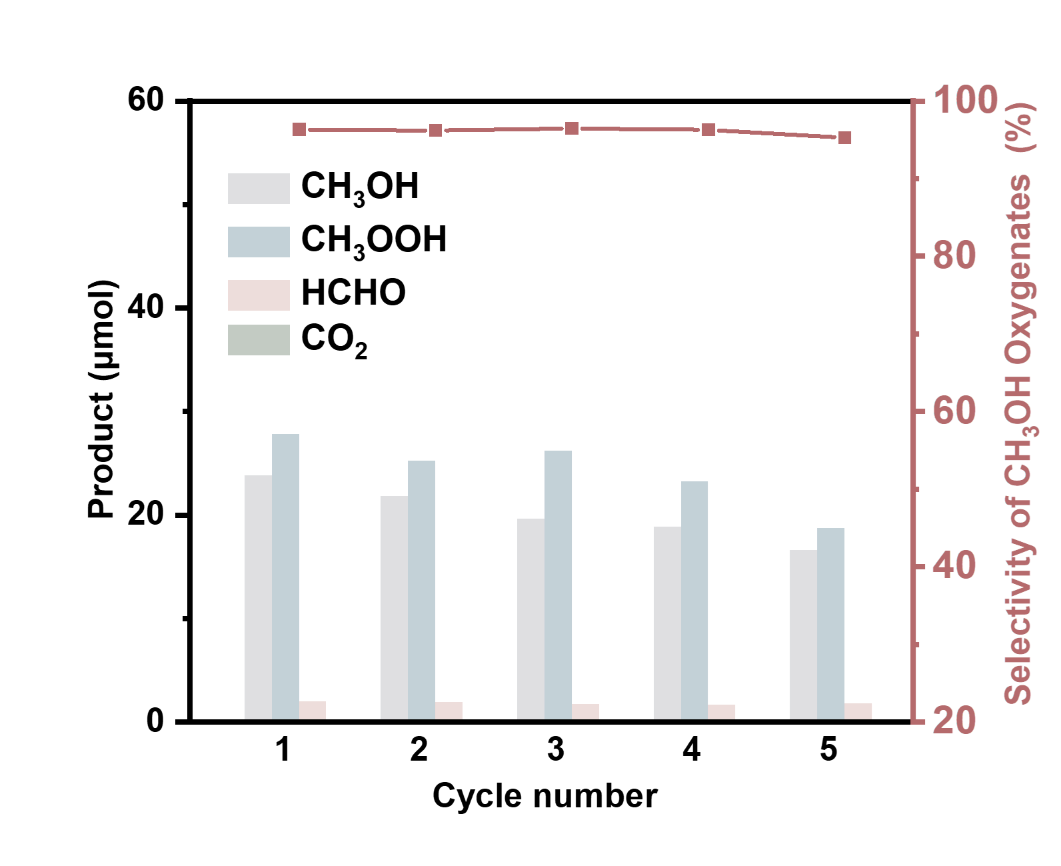


**Figure S9.** Recyclability of Def-CuCN/NU in photocatalytic oxidation of CH_4_.


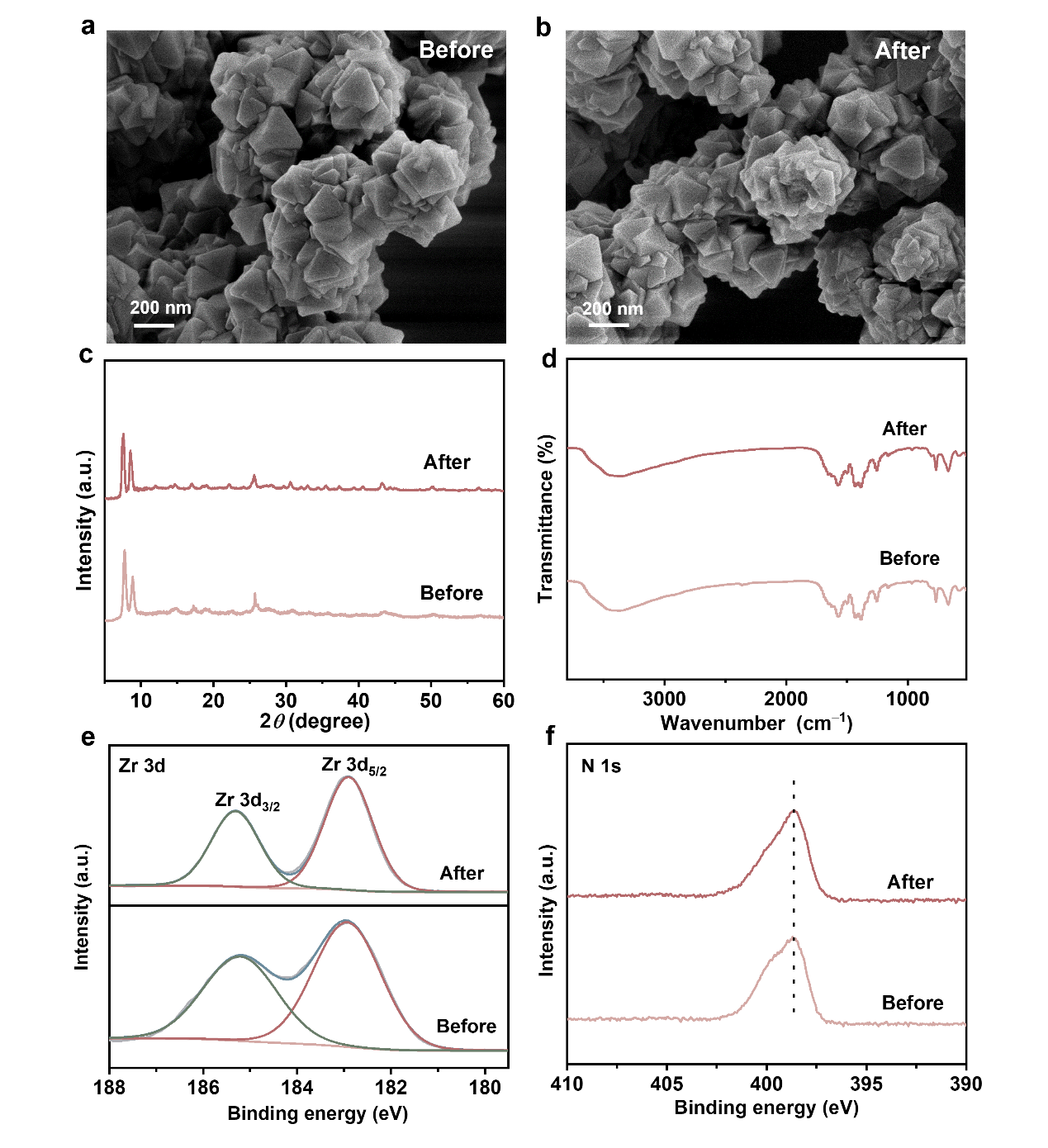


**Figure S10.** Characterizations of Def-CuCN/NU before and after reaction: (a, b) SEM images; (c) XRD patterns; (d) FT-IR spectra; (e) XPS spectra of Zr 3d; (f) XPS spectra of N 1s.

**Figure S11.** Time-resolved PL decay spectra of CuCN/NU and Def-CuCN/NU.


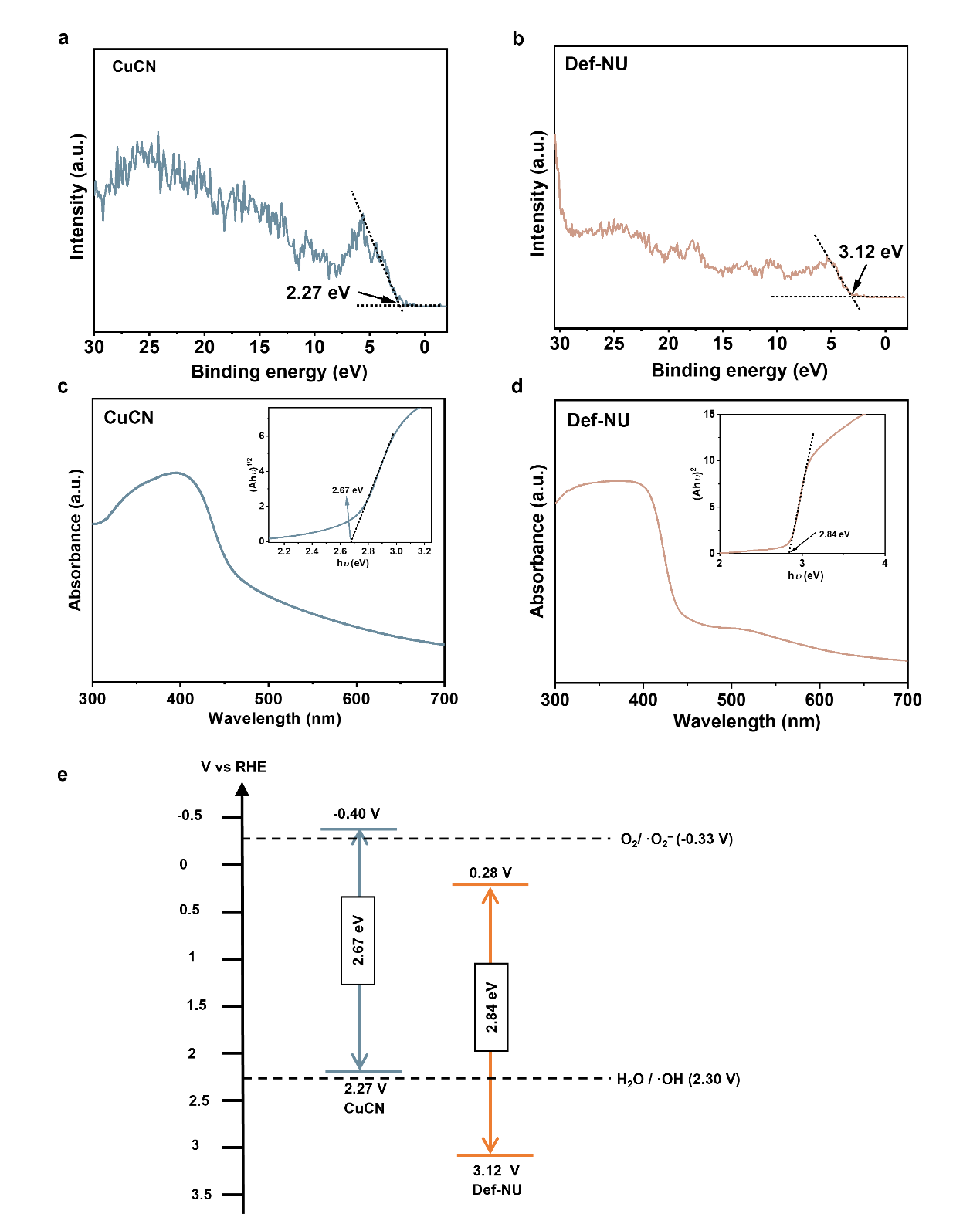


**Figure S12.** (a, b) XPS valence band spectrum, (c, d) UV−vis diffuse reflectance spectra and corresponding Tauc plots (insets), and (e) schematic band structure diagrams of CuCN and Def-NU.


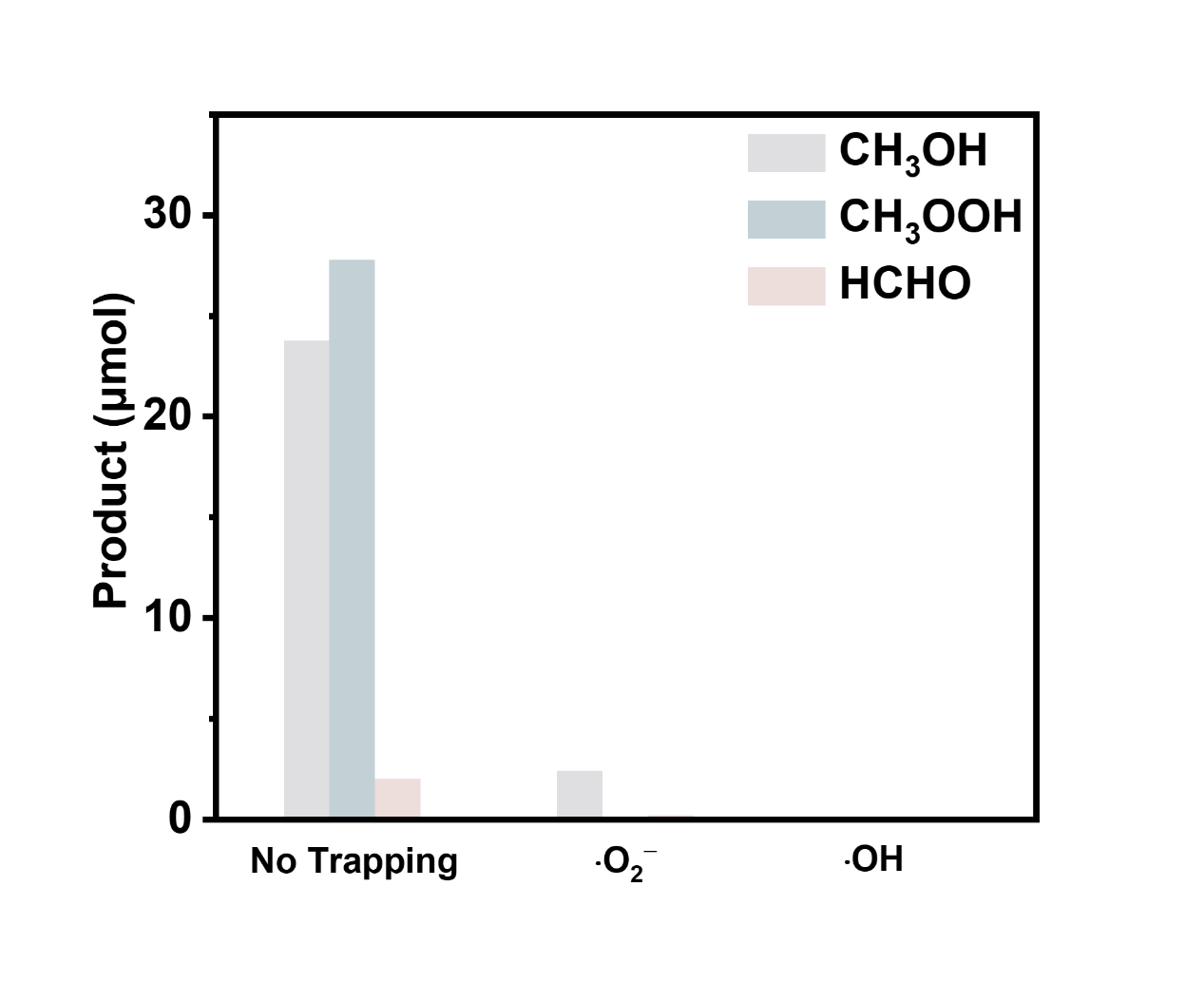


**Figure S13*.*** The effects of the trapping agents of para-benzoquinone (for ·O_2_^−^ radicals) and salicylic acid (for ·OH radicals) on the production of methanol oxygenates over Def-CuCN/NU.

**Table S1** The *S*_BET_, *V*_pore_, and *d*_pore_ of CuCN, NU, CuCN/NU, and Def-CuCN/NU.

| Sample | *S*_BET_  (m^2^ g^−1^) | *V*_pore_  (cm^3^ g^−1^) | *d*_pore_  (nm) |
| --- | --- | --- | --- |
| CuCN | 6 | 0.02 | 17.7 |
| NU | 841 | 0.37 | 2.0 |
| CuCN/NU | 519 | 0.24 | 2.4 |
| Def-CuCN/NU | 749 | 0.35 | 3.2 |

**Table S2** XPS results derived COO^−^/Zr peak area ratio of CuCN/NU and Def-CuCN/NU.

| Sample | COO^−^ Peak Area | Zr 3d_5/2_ Peak Area | COO^−^/Zr ratio |
| --- | --- | --- | --- |
| CuCN/NU | 29218 | 70478 | 41.4 |
| Def-CuCN/NU | 6875 | 64356 | 10.7 |

**Table S3** Control experiments of photocatalytic oxidation of CH_4_ to methanol oxygenates over the Def-CuCN/NU catalyst.^a^

| Entry | Reaction parameter | Methanol oxygenates (µmol) |
| --- | --- | --- |
| 1 | Dark | n.d.^b^ |
| 2 | No CH_4_ | n.d. |
| 4 | No catalyst | n.d. |

^a^ Reaction conditions: 10 mg of catalyst, 60 mL of distilled H_2_O, 19 bar of CH_4_, 1 bar of O_2_, 25°C, 300 < *λ* < 780 nm, light intensity of ca. 200 mW cm^−2^, stirring rate of 800 rpm, and reaction time of 3 h. For reaction condition investigation, only the specified parameter was varied.

**Table S4** Comparison with the catalytic performances of some literature catalysts in photocatalytic oxidation of CH_4_ to methanol oxygenates

| Catalyst | | Oxidant | Reaction condition | Productivity of Methanol oxygenates (μmol g^−1^ h^−1^) | Selectivity of methanol oxygenates (%) | Ref. |
| --- | --- | --- | --- | --- | --- | --- |
| Def-CuCN/NU | | O_2_ | 19 bar CH_4_, 1 bar O_2_, 25 ºC | 1718 | 96.5 | This work |
| Ru_1_O*_x_*/ZnO | | O_2_ | 20 bar CH_4_, 1 bar O_2_, 25 ºC | 321 | 90.9 | [1] |
| Au*_x_*/ZnO | | O_2_ | 15 bar CH_4_, 5 bar O_2_, 30 ºC | 1371 | 99.1 | [2] |
| Pd/H-TiO_2_ | | O_2_ | 2 MPa, CH_4_:O_2_ 50:1, 45 ºC | 2400 | 54.8 | [3] |
| Ni-NC/TiO_2_ | | O_2_ | 20 bar CH_4_, 1 bar O_2_, 25ºC | 2100 | 39.2 | [4] |
| Au-CoO*_x_*/TiO_2_ | O_2_ | | 20 bar CH_4_, 1 bar O_2_, 25 ºC | 2540 | 91 | [5] |
| q-BiVO_4_ | O_2_ | | 10 bar CH_4_, 10 bar O_2_, 25 ºC | 370 | 96.6 | [6] |
| Au1/BP | O_2_ | | 30 bar CH_4_, 3 bar O_2_, 90 ºC | 57 | 99.0 | [7] |
| Au_1.0_Fe_0.33_- ZnO | O_2_ | | 18 bar CH_4_, 2 bar O_2_, 20 ºC | 1400 | 93 | [8] |
| Au NPs/In_2_O_3_ | O_2_ | | 20 bar CH_4_, 10 bar O_2_, 20 ºC | 2030 | 89 | [9] |
| ZnO | O_2_ | | 19 bar CH_4_, 2 bar O_2_, 30 ºC | 820 | 74.3 | [10] |
| Au/ZnO | O_2_ | | 19 bar CH_4_, 2 bar O_2_, 30 ºC | 1700 | 68.5 | [10] |

**References**

[1] H. Gong, L. Zhang, C. Deng, M. Liu, X. Liu, Y. Huang, K. Zhou, P. He, J. Li, Y. Yang, L. Wang, Q. Yang, Z. Bao, Q. Ren, T. Tan, S. Yao, Z. Zhang, *J. Am. Chem. Soc.* **2025**, 147, 9134.

[2] W. Zhou, X. Qiu, Y. Jiang, Y. Fan, S. Wei, D. Han, L. Niu, Z. Tang, *J. Mater. Chem. A* **2020**, 8, 13277.

[3] X. Zhang, Y. Wang, K. Chang, S. Yang, H. Liu, Q. Chen, Z. Xie, Q. Kuang, *Appl. Catal. B* **2023**, 320, 121961.

[4] H. Song, H. Huang, X. Meng, Q. Wang, H. Hu, S. Wang, H. Zhang, W. Jewasuwan, N. Fukata, N. Feng, J. Ye, *Angew. Chem. Int. Ed.* **2023**, 62, e202215057.

[5] H. Song, X. Meng, S. Wang, W. Zhou, S. Song, T. Kako, J. Ye, *ACS Catal.* **2020**, 10, 14318.

[6] Y. Fan, W. Zhou, X. Qiu, H. Li, Y. Jiang, Z. Sun, D. Han, L. Niu, Z. Tang, *Nat. Sustain.* **2021**, 4, 509.

[7] L. Luo, J. Luo, H. Li, F. Ren, Y. Zhang, A. Liu, W.-X. Li, J. Zeng, *Nat. Commun.* **2021**, 12, 1218.

[8] H. Du, X. Li, Z. Cao, S. Zhang, W. Yu, F. Sun, S. Wang, J. Zhao, J. Wang, Y. Bai, J. Yang, P. Yang, B. Jiang, H. Li, *Appl. Catal. B* **2023**, 324, 122291.

[9] Y. Jiang, S. Li, S. Wang, Y. Zhang, C. Long, J. Xie, X. Fan, W. Zhao, P. Xu, Y. Fan, C. Cui, Z. Tang, *J. Am. Chem. Soc.* **2023**, 145, 2698.

[10] Z. Xiao, Z. Wan, J. Zhang, J. Jiang, D. Li, J. Shen, W. Dai, Y. Li, X. Wang, Z. Zhang, *ACS Catal.* **2024**, 14, 9104.
